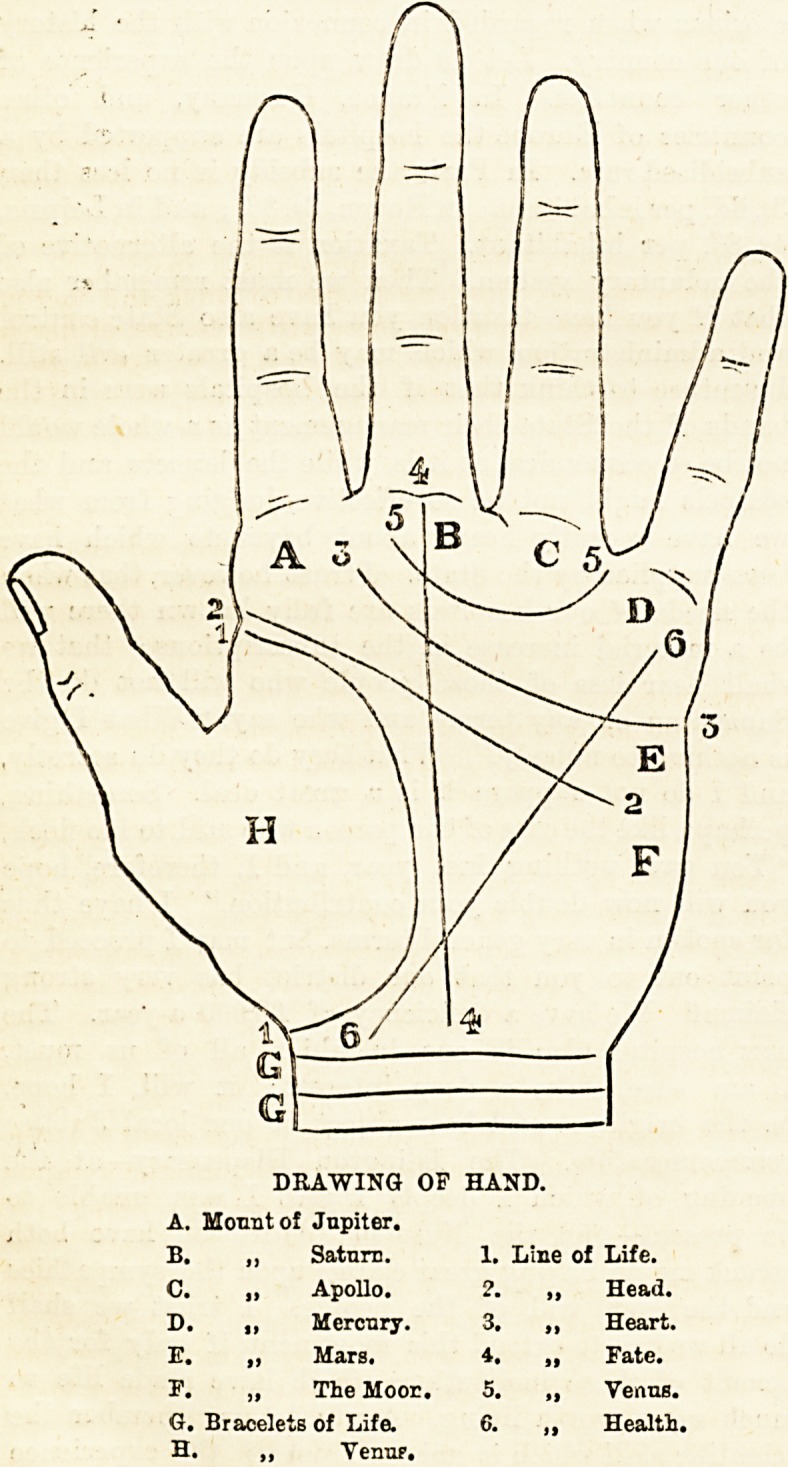# Palmistry Notes

**Published:** 1887-12-31

**Authors:** 


### Amusements for Convalescents.

### PALMISTRY NOTES.

By a Lady.

(*Continued from page* 211.)

DRAWING OF HAND.

| | | | |
|---|---|---|---|
| A. | Mount of Jupiter. | | |
| B. | ,, Saturn. | 1. | Line of Life. |
| C. | ,, Apollo. | 2. | ,, Head. |
| D. | ,, Mercury. | 3. | ,, Heart. |
| E. | ,, Mars. | 4. | ,, Fate. |
| F. | ,, The Moon. | 5. | ,, Venus. |
| G. | Bracelets of Life. | 6. | ,, Health. |
| H. | ,, Venus. | | |

**Mounds of the Palm**—(*continued*).

*The Mount of Mercury* (D) gives us business capacity, hrewdness, perseverance, and scientific ability. We shall be intelligent, quick in expedient, fond of argument and eloquent, good at out-door games and sports, fond of children, and, on the whole, good humoured. Barristers and clergymen should have Mercury developed. Many lines on the mount indicate scientific gifts, but mere little rays and flecks show a chattering disposition. One firm line, unexpected good fortune. A line on the mount joined to a line on the edge of the hand indicates a love affair of short duration, ending in death. A line on the mount touching the heart-line shows generosity. Lines on the edge of the hand continuing to Mercury denote the number of attachments we have had. One clear line shows a deep and lasting affection; many lines, numerous small and not very serious "fancies." A spot denotes misfortune through speculation. *The Mount of Mars* (E) smooth and unrayed gives energy and determination, capacity to lead, patience under difficulties, resource in emergencies, and great self-control. A much-rayed Mount of Mars, with short nails indicates aggressiveness and combativeness, both serious obstacles to our success in life. A cross on the mount shows danger arising from the exercise of these characteristics. Absence of the mount denotes weakness and indecision.

Just below the Mount of Mars comes the *Mount of the Moon* (F); if well developed we shall be poetic and imaginative, indolent, and fond of variety in our friends and surroundings. *Restless* rather than *active*, our love of change will make us incapable of any sustained effort. Much given to caprice, especially in our love affairs, which are distinguished by admiration for those whose difference in position or age make them unsuitable as partners for life. Many lines on the mount indicate constant discontent with one's surroundings, and much rayed and crossed, fretfulness and over anxiety. A straight line from Mercury to the Moon indicates good fortune. Lines from the wrist straight on to the moon indicate journeys by water, and a star on one of these voyage lines danger of death by drowning.

We have now come to the last mount—*Venus* (H). This mount developed gives us love of pleasure, melody, beauty, affability, with a talent for music, painting, and poetry; great sociability, and love of appreciation and admiration. A number of lines on the mount indicate warmth of temperament. A line from the mount to Mercury indicates wealth and affection. Three lines from the mount on to Jupiter (A) denote happiness and generosity.

In studying the "mounds" it is well to remember that the one most conspicuous in a hand is the one which gives the *tone* to the whole character. It is, however, rarely that one mount only is prominent; usually two or three other mounts are specially noticeable, and then we find a blending o characteristics which will modify the too-powerful development of the prevailing mount. Sometimes a mound is broad and full, instead of high, spreading to an adjoining mound. In this case it will partake of the qualities of the mount whose place it has usurped. The *Lines of the Palm* will form the subject of my next article.

(*To be continued.*)

## Figures and Tables

**Figure f1:**